# Supplementary material for: Exploring the potential of Schmidtea mediterranea as a model for reproductive toxicity through analysis of sterility caused defective m6A RNA regulation
Source: Curr Res Toxicol. 2025 Oct 25;9:100262. doi: 10.1016/j.crtox.2025.100262 (PMC12661160; doi:10.1016/j.crtox.2025.100262)

SUPPLEMENTARY FIGURE LEGENDS

Supplementary Figure S1. Neoblasts and GSCs were not affected by perturbation of m^{6}A methylation pathway component expression in asexual planarians. Neoblasts (marked by *gH4*) and presumptive germline stem cells (marked by *nanos and gH4*) were present at comparable levels in control RNAi samples and after more than four weeks of m^{6}A writer and reader gene knockdown. Fraction of samples showing phenotypes undistinguishable from controls is shown in parenthesis. Scale bar: 50 μm.

Supplementary Figure S2. No defects in planarian regeneration were observed upon m^{6}A methyltransferase gene knockdown. (A-B) Normal regeneration is observed in trunk (A) and tail (B) fragments of planarians subjected to RNAi-mediated disruption of *Smed-METTL3* expression, *Smed-METTL14* expression, or *Smed-METTL3;Smed-METTL14* simultaneous knockdown. DAPI staining of cell nuclei (gray) is shown along with visualization of the brain and ventral nerve cords labeled using anti-SYNORF antibodies (green). Brackets show regenerated head or tail. Asterisk indicates position of the pharynx. Scale bar is 500 μm.

Supplementary Figure S3. No decrease in fission events observed during m^{6}A methyltransferase gene knockdown. Graph portraying cumulative fission events observed during knockdown of writer complex genes (*METTL3;METTL14(RNAi)* or *WTAP(RNAi)*) and reader genes (*YTHDF1-1(RNAi)*, *YTHDF2-3(RNAi)*, *YTHDF2-2(RNAi)*, *YTHDF1-2(RNAi)*, or *YTHDF2-1(RNAi)*).

Tasaki_Fig_S1

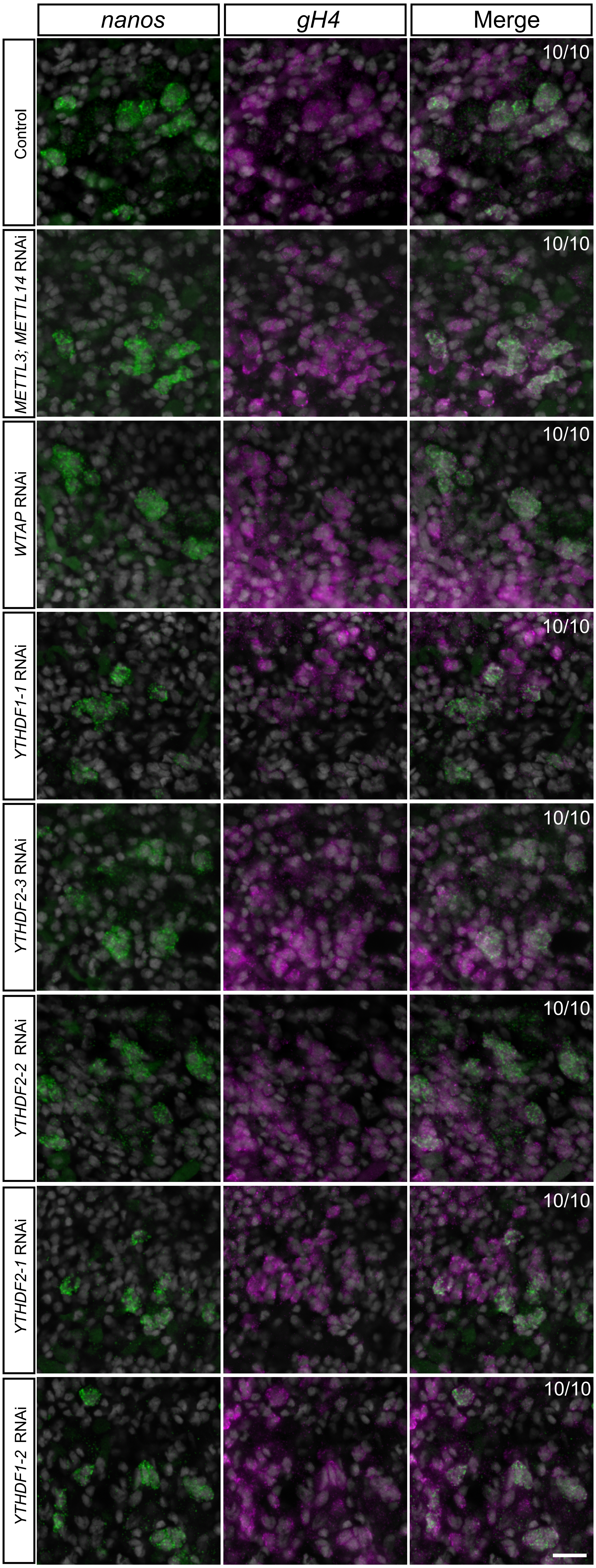

Tasaki_Fig_S2

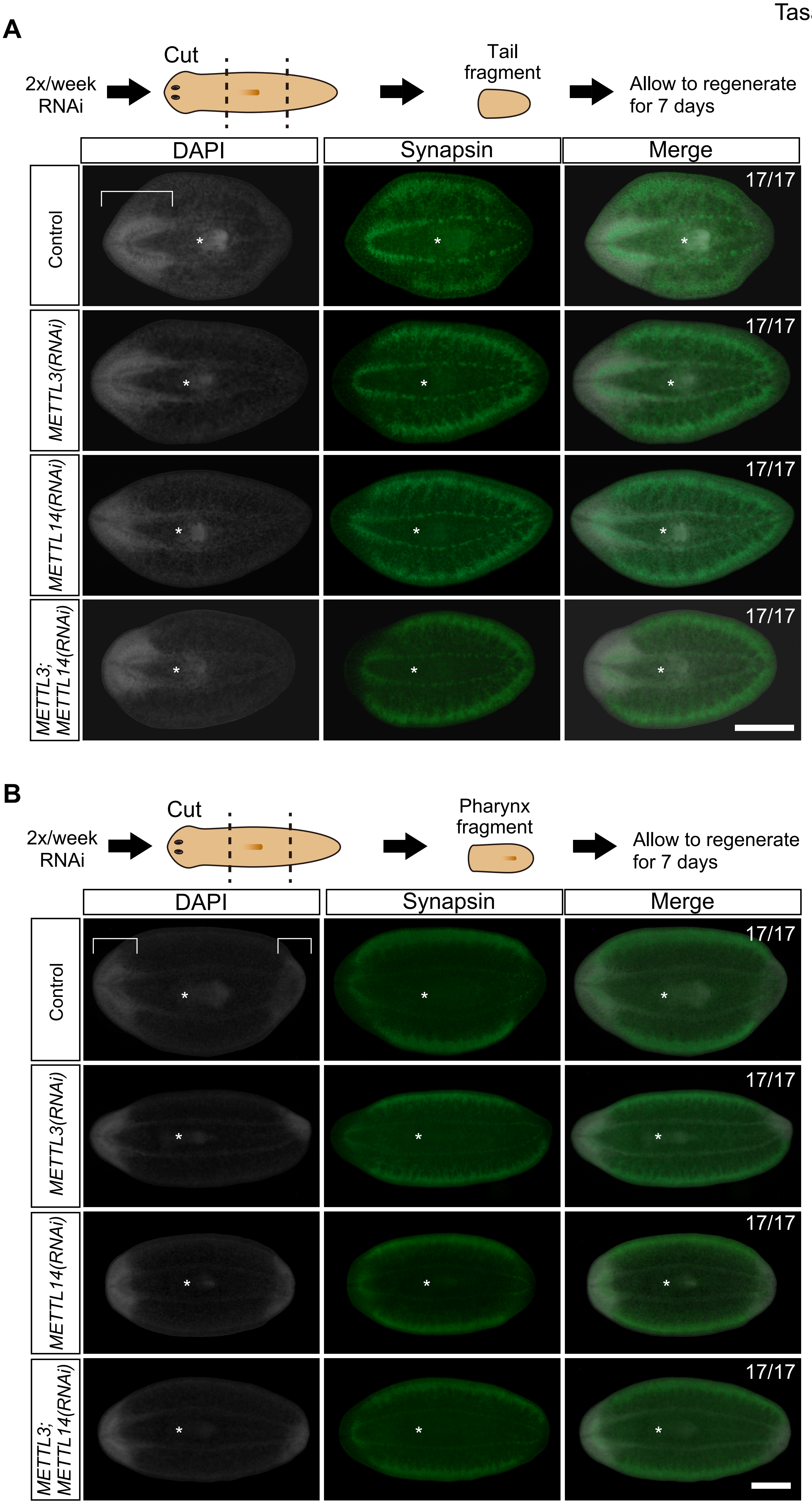

Tasaki_Fig_S3

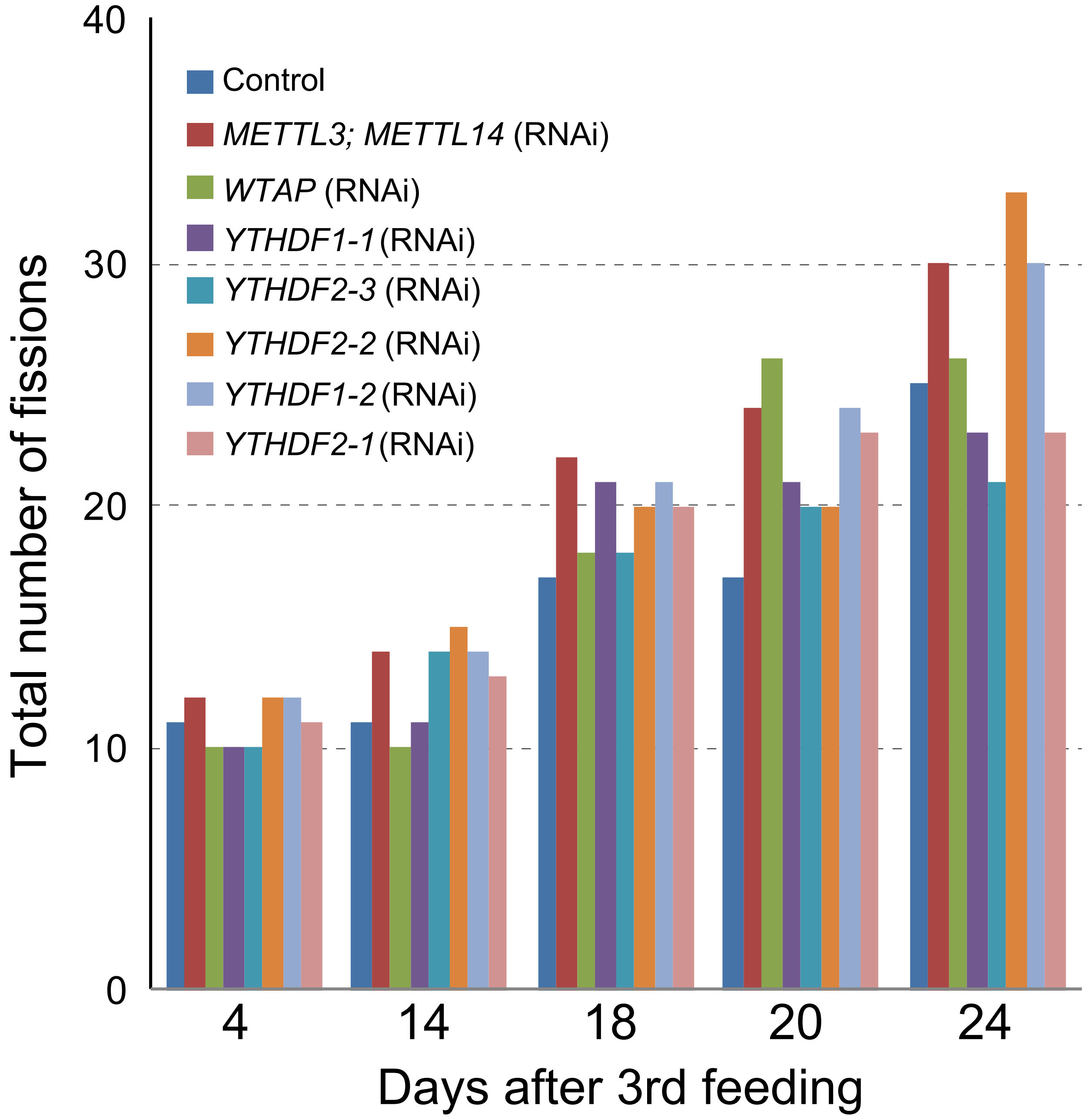

Supplement: Supplementary Data 1 [file mmc1.pdf]
